# Comparing the clinical outcomes of initial surgery and primary definitive radiotherapy with a dosage of 6600 cGy or higher in cT1−2N0M0 oral cavity squamous cell carcinoma: A nationwide cohort study

**DOI:** 10.1002/cam4.7127

**Published:** 2024-05-21

**Authors:** Chien‐Yu Lin, Wen‐Cheng Chen, Yu‐Wen Wen, Kang‐Hsing Fan, Jin‐Ching Lin, Shu‐Hang Ng, Yao‐Te Tsai, Shu‐Ru Lee, Chung‐Jan Kang, Li‐Yu Lee, Chih‐Yen Chien, Chun‐Hung Hua, Cheng Ping Wang, Tsung‐Ming Chen, Shyuang‐Der Terng, Chi‐Ying Tsai, Hung‐Ming Wang, Chia‐Hsun Hsieh, Chih‐Hua Yeh, Chih‐Hung Lin, Chung‐Kan Tsao, Nai‐Ming Cheng, Tuan‐Jen Fang, Shiang‐Fu Huang, Li‐Ang Lee, Ku‐Hao Fang, Yu‐Chien Wang, Wan‐Ni Lin, Li‐Jen Hsin, Tzu‐Chen Yen, Chun‐Ta Liao

**Affiliations:** ^1^ Department of Radiation Oncology Chang Gung Memorial Hospital and Chang Gung University Taoyuan Taiwan, ROC; ^2^ Clinical Informatics and Medical Statistics Research Center Chang Gung University Taoyuan Taiwan, ROC; ^3^ Division of Thoracic Surgery Chang Gung Memorial Hospital Taoyuan Taiwan, ROC; ^4^ Department of Radiation Oncology Changhua Christian Hospital Changhua Taiwan, ROC; ^5^ Department of Diagnostic Radiology Chang Gung Memorial Hospital and Chang Gung University Taoyuan Taiwan, ROC; ^6^ Department of Otorhinolaryngology‐Head and Neck Surgery Chang Gung Memorial Hospital Chiayi Taiwan, ROC; ^7^ Research Service Center for Health Information Chang Gung University Taoyuan Taiwan, ROC; ^8^ Department of Otorhinolaryngology, Head and Neck Surgery Chang Gung Memorial Hospital and Chang Gung University Taoyuan Taiwan, ROC; ^9^ Department of Pathology Chang Gung Memorial Hospital and Chang Gung University Taoyuan Taiwan, ROC; ^10^ Department of Otolaryngology, Chang Gung Memorial Hospital Kaohsiung Medical Center Chang Gung University College of Medicine Taoyuan Taiwan, ROC; ^11^ Department of Otorhinolaryngology China Medical University Hospital Taichung Taiwan, ROC; ^12^ Department of Otolaryngology National Taiwan University Hospital and College of Medicine Taipei Taiwan, ROC; ^13^ Department of Otolaryngology, Shuang Ho Hospital Taipei Medical University New Taipei City Taiwan, ROC; ^14^ Department of Head and Neck Surgery Koo Foundation Sun Yat‐Sen Cancer Center Taipei Taiwan, ROC; ^15^ Department of Oral and Maxillofacial Surgery, Chang Gung Memorial Hospital Chang Gung University Taoyuan Taiwan, ROC; ^16^ Department of Medical Oncology Chang Gung Memorial Hospital and Chang Gung University Taoyuan Taiwan, ROC; ^17^ Department of Plastic and Reconstructive Surgery Chang Gung Memorial Hospital and Chang Gung University Taoyuan Taiwan, ROC; ^18^ Department of Nuclear Medicine and Molecular Imaging Center Chang Gung Memorial Hospital and Chang Gung University Taoyuan Taiwan, ROC

**Keywords:** cancer registry, clinical outcomes, cT1−2N0M0, definite radiotherapy, oral cavity squamous cell carcinoma, surgery

## Abstract

**Background:**

To compare the clinical outcomes of two treatment modalities, initial surgery and primary definitive radiotherapy (RT), in Taiwanese patients diagnosed with cT1−2N0M0 oral cavity squamous cell carcinoma (OCSCC).

**Methods:**

Between 2011 and 2019, we analyzed data for 13,542 cT1−2N0M0 patients who underwent initial surgery (*n* = 13,542) or definitive RT with a dosage of at least 6600 cGy (*n* = 145) for the treatment of OCSCC. To account for baseline differences, we employed propensity score (PS) matching, resulting in two well‐balanced study groups (initial surgery, *n* = 580; definitive RT, *n* = 145).

**Results:**

Before PS matching, the 5‐year disease‐specific survival (DSS) rates were 88% for the surgery group and 58% for the RT group. After PS matching, the 5‐year DSS rates of the two groups were 86% and 58%, respectively. Similarly, the 5‐year overall survival (OS) rates before PS matching were 80% for the surgery group and 36% for the RT group, whereas after PS matching, they were 73% and 36%, respectively. All these differences were statistically significant (*p* < 0.0001). A multivariable analysis identified treatment with RT, older age, stage II tumors, and a higher burden of comorbidities as independent risk factors for both DSS and OS. We also examined the 5‐year outcomes for various subgroups (margin ≥5 mm, margin <5 mm, positive margins, RT combined with chemotherapy, and RT alone) as follows: DSS, 89%/88%/79%/63%/51%, respectively, *p* < 0.0001; OS, 82%/79%/68%/39%/32%, respectively, *p* < 0.0001.

**Conclusions:**

In Taiwanese patients with cT1−2N0M0 OCSCC, a remarkably low proportion (1.1%) completed definitive RT. A significant survival disparity of 30% was observed between patients who underwent initial surgery and those who received definitive RT. Interestingly, even patients from the surgical group with positive surgical margins exhibited a significantly superior survival compared to those in the definitive RT group.

## Graphical abstract

Patients with cT1−2N0M0 oral cavity cancer who underwent initial surgery demonstrated significantly improved disease‐specific and overall survival rates compared to those who received primary definitive radiotherapy (≥6600 cGy), even after propensity score matching. Subgroup analyses further revealed that survival outcomes remained significantly better for the positive margin subgroup compared to the radiotherapy group, irrespective of whether they received concurrent chemotherapy.

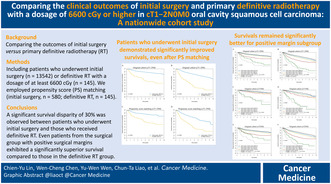

## 1 INTRODUCTION

The primary therapeutic strategy for oral cavity squamous cell carcinoma (OCSCC) is surgical excision, supplemented by adjuvant therapy tailored to post‐operative pathological risk factors (RFs).^1^ According to the National Comprehensive Cancer Network (NCCN) guidelines, initial surgery is the preferred treatment for OCSCC cases classified as cT1−2N0M0. However, these guidelines also propose definitive radiotherapy (RT) as an alternative therapeutic approach for this patient group.^1^ It is important to note that in the context of resected OCSCC, positive margins are considered an unfavorable pathological feature. If re‐resection is not feasible, a combination of systemic treatment and RT should be pursued.^1^ Currently, no clinical trials have been conducted to directly compare the outcomes of initial surgery and definitive RT in patients with cT1−2N0 OCSCC. This lack of head‐to‐head comparison could be due to the significant outcome differences observed in these patients. Specifically, around a 30% survival disparity has been noted between patients who undergo initial surgery and those who receive definitive RT.^2^, ^3^, ^4^ Despite this, it is worth noting that approximately 5% of patients still opt for non‐surgical treatments. This choice may be influenced by patient‐related factors such as advanced age and existing comorbidities, or hospital‐related variables like the complexities associated with certain conditions, including retromolar trigone tumors.^2^, ^3^ Numerous nationwide studies have consistently shown that initial surgical intervention yields superior outcomes compared to definitive RT. However, the selection criteria for the RT group in these studies frequently lacked rigorousness, and not all patients were administered an effective definitive RT dose ≥6600 cGy.^1^, ^2^, ^3^, ^4^ Consequently, the survival disparity between patients who initially underwent surgery and those who received definitive RT of at least 6600 cGy may not be as significant as previously thought. While definitive RT can provide moderate survival benefits, it also introduces the risk of certain morbidities.^5^ Notably, a study from the National Cancer Database (NCDB) on initial surgery for the cT1−2N0 patient group showed that approximately 20% of patients required postoperative adjuvant therapy. This was due to factors such as tumor upstaging, including occult nodal metastasis, or the presence of adverse pathological features.^3^

Currently, there is a scarcity of comprehensive investigations comparing the clinical outcomes of initial surgery and definitive RT with a dose of at least 6600 cGy for patients with cT1−2N0M0 OCSCC. In this nationwide study conducted in Taiwan, our objectives were to: (1) determine the percentage of patients who received definitive RT, (2) compare survival outcomes between the initial surgery and definitive RT groups, (3) identify prognostic factors, and (4) assess the prognosis of the surgical subgroup with positive margins in comparison to the definitive RT group.

## 2 METHODS

### 2.1 Data sources

The current study utilized data from the Taiwan Cancer Registration Database (TCRD) “long‐form,” a nationwide digital dataset that provides comprehensive longitudinal information on cancer stage, disease recurrences, and treatments received by patients diagnosed with cancer in major Taiwanese hospitals. However, the TCRD does not include information on chemotherapy regimens and salvage therapy for individuals experiencing disease relapse. Survival outcomes were sourced from the Taiwanese National Health Insurance Research Dataset (TNHIRD). The study results were presented in accordance with the guidelines established by the Reporting Recommendations for Tumor Marker Prognostic Studies (REMARK).^6^, ^7^ The research protocol was approved by the Ethics Committee of the Chang Gung Memorial Hospital (reference number: 201801398B0A3), which also issued a waiver for acquiring written informed consent.

### 2.2 Treatment protocol and follow‐up schedule

Patients with OCSCC face variable outcomes, which are heavily influenced by the surgical approach and adjuvant therapy. Therefore, a comprehensive strategy for decision‐making, treatment, clinical management, and follow‐up is crucial in regions where betel quid chewing is prevalent. Recognizing this, Taiwanese hospitals specializing in OCSCC treatment initiated the implementation of a multidisciplinary team care approach in January 2004. In general, the treatment and follow‐up protocols, including adjuvant therapy based on pathological RFs, adhered to the NCCN guidelines.^1^

In the treatment plan for stages I and II oral cancers, the choice to use preventive nodal irradiation is influenced by the primary tumor's anatomical position and the patient's overall health status. Tumors located more than 2 cm from the center line are less likely to metastasize to the lymph nodes on the opposite side. In these cases, particularly for smaller tumors in the cheek lining or behind the molars, preventive radiation is typically applied to the lymph nodes on the same side of the upper neck. However, for tumors located centrally, like those on the tongue, floor of the mouth, or hard palate, a thorough preventive radiation of the lymph nodes on both sides of the upper neck is necessary. Nonetheless, treatment plans can be adjusted for older patients or those with poor general conditions to exclude preventive nodal radiation, thus minimizing the risk of side effects from the treatment.

### 2.3 Patient selection

**FIGURE 1:**
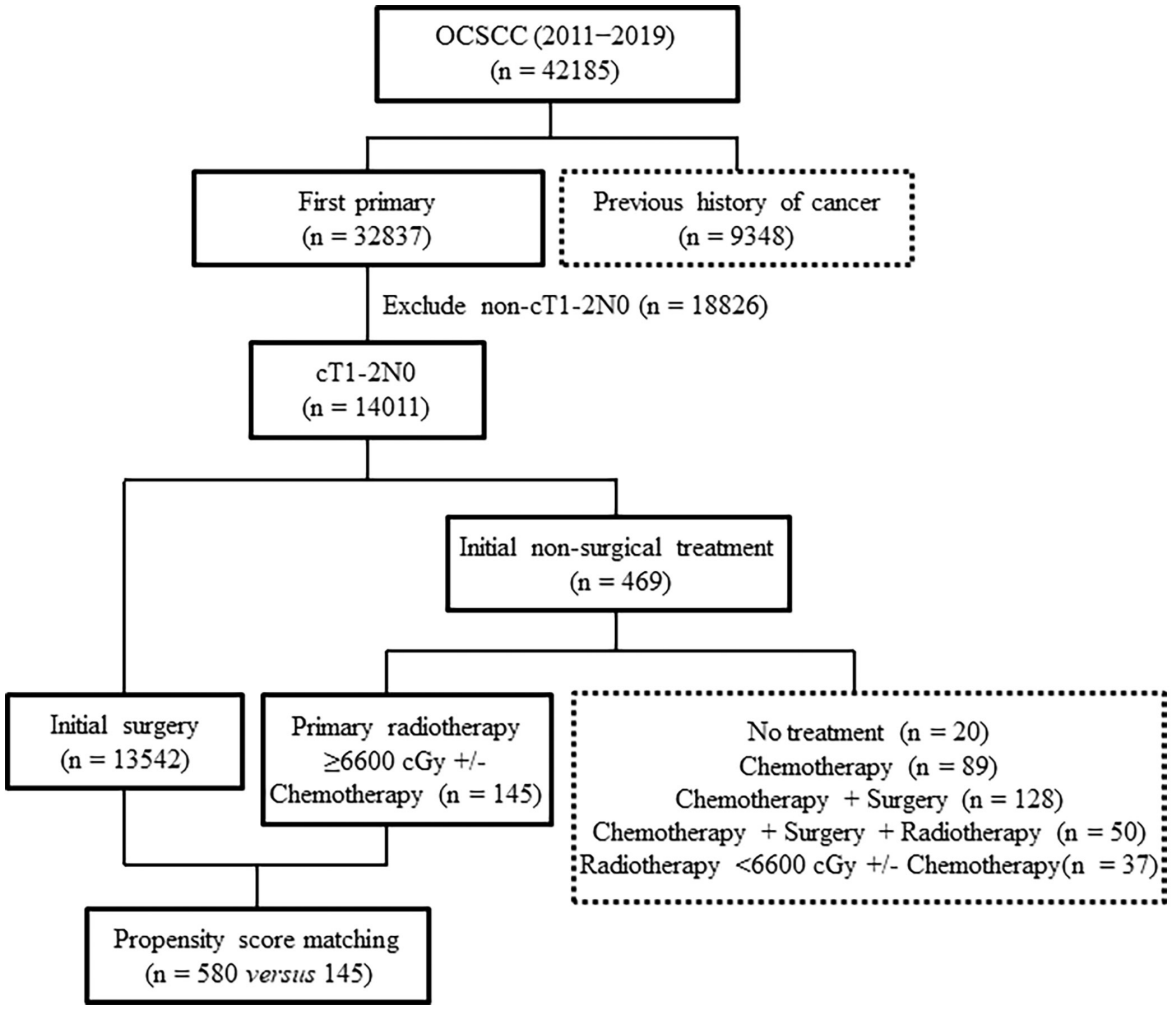
Flow of patients through the study.

The study focused on patients diagnosed with OCSCC between 2011 and 2019, totaling 42,185 individuals. The selection of cases was based on specific codes from the International Classification of Diseases for Oncology, Third Edition (ICD‐O‐3). These included various forms of OCSCC such as lip [C00.3; C00.4; C00.5], tongue [C02.0; C02.1; C02.2; C02.3], gum [C03.0; C03.1; C03.9], floor of mouth [C04.0; C04.1; C04.8; C04.9], hard palate [C05.0], cheek [C06.0], retromolar area [C06.2], and other forms of oral cavity cancer [C06.1; C06.8; C06.9]. After excluding patients with a prior history of malignancies (*n* = 9348) and those with non‐cT1−2N0 tumors (*n* = 18,826), the study initially collected data on 14,011 patients with cT1−2N0 OCSCC. Of these, 13,542 (96.7%) underwent initial surgery, whereas 469 (3.3%) received non‐surgical treatment approaches. Out of the initial non‐surgical group (*n* = 469), we excluded those who did not receive any treatment (*n* = 20), those who were treated with chemotherapy alone (*n* = 89), those who underwent chemotherapy combined with non‐initial surgery (*n* = 128), those who received a combination of chemotherapy, non‐initial surgery, and RT (*n* = 50), and patients who received RT with a dosage of less than 6600 cGy (*n* = 37). As a result, the final study cohort consisted of 13,687 patients. Among them, 13,542 (98.9%) underwent initial surgery, whereas 145 (1.1%) received definitive RT with a dose greater than or equal to 6600 cGy. Figure 1 illustrates the patient flow within the study. The follow‐up period was determined by measuring the time from treatment initiation to either the patient's demise or the study completion in December 2020.

### 2.4 Data collection

The research variables were gathered from the 2019 release of the TCRD and the 2020 release of the TNHIRD. Data analysis was performed in August 2023. The TCRD adheres to the guidelines specified in the Standards for Oncology Registry Entry (STORE) manual.^8^ Pertinent information regarding morbidity and mortality related to OCSCC was extracted from the cause‐specific database within the TNHIRD. Subsequently, these data were utilized to compute disease‐specific survival (DSS) and overall survival (OS).

### 2.5 Statistical analysis

In order to mitigate bias arising from baseline differences between patients undergoing initial surgery or receiving definitive RT, we employed propensity score (PS) matching via logistic regression. This model scrutinized both treatment approaches, taking into account potential confounding factors such as tumor subsite, sex, clinical stage, and Charlson Comorbidity Index (CCI). We then compared clinical outcomes in a 4:1 matched sample (initial surgery vs. primary RT), which was based on the estimated propensity scores. Following PS matching, all covariates demonstrated standardized mean differences (SMDs) of less than 0.1, suggesting a satisfactory balance between the groups. To visualize survival estimates, we generated Kaplan–Meier plots and used the log‐rank test for statistical comparison. To investigate the relationships between the variables under study and survival outcomes, we performed both univariable and multivariable Cox proportional hazards regression analyses. We utilized a stepwise selection method, integrating all variables from the univariable analysis into the multivariable model. Given the potential breach of the non‐informative censoring assumption for DSS, we also applied a competing risk model. The findings are expressed as hazard ratios (HRs) with their corresponding 95% confidence intervals (CIs). All statistical tests were two‐sided and conducted at a 5% significance level.

**FIGURE 2:**
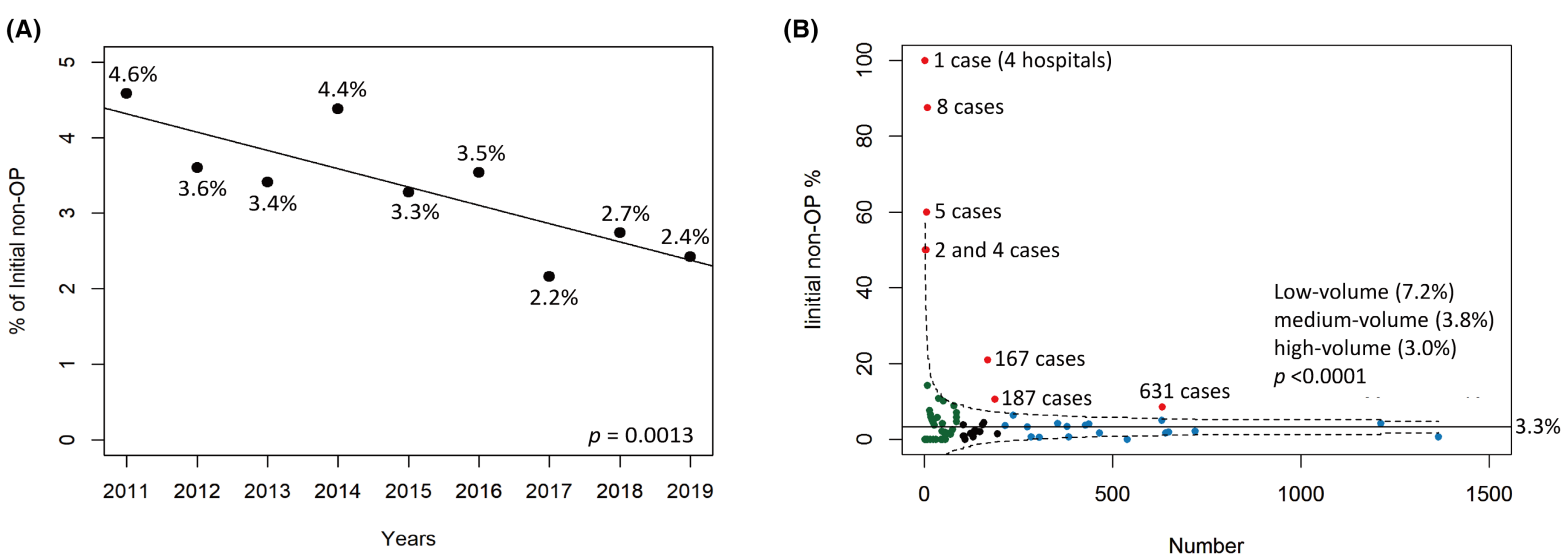
Annual incidence of initial non‐surgical treatment (A) and distribution of 143 specialized oral cavity cancer hospitals, classified by the volume of treated cases and the incidence of initial non‐surgical treatment (B). Each data point signifies the proportion of initial non‐surgical treatments observed at various hospitals. Green dots signify hospitals treating 1–99 cases, black dots represent those handling 100–199 cases, and blue dots correspond to institutions managing ≥200 cases. The solid line illustrates the mean value, which is determined by dividing the aggregate number of initial non‐surgical procedures by the entire sample size. Meanwhile, the dashed lines depict the upper control limit and lower control limits. Data points exceeding the upper control limit are denoted by red dots.

For the visualization and assessment of non‐surgical treatment rates across various hospitals (Figure 2), we employed a control *p*‐chart process.^9^ The center line (CL) of the *p*‐chart represents the mean value, whereas the upper control limits (UCL) and lower control limits (LCL) were determined using the following specific equations, where $\bar{p}$ denotes the CL:

UCL_*p*_ $=\bar{p}+3\sqrt{\frac{\bar{p}\;\left(1-\bar{p}\right)}{n_{i}}\;}$, ith sample is of size $n_{i}$.

LCL_*p*_ =$\;\bar{p}-3\sqrt{\frac{\bar{p}\;\left(1-\bar{p}\right)}{n_{i}}\;}$, ith sample is of size $n_{i}$.

## 3 RESULTS

### 3.1 Yearly prevalence of non‐surgical care and usage rates in Taiwanese hospitals

In the original cohort of cT1–2N0 patients—which included 14,011 cases—a non‐surgical treatment approach was initially adopted for 3.3% (469 out of 14,011) of the participants. The yearly breakdown of patients who received non‐surgical treatment first was as follows: 4.6% in 2011, 3.6% in 2012, 3.4% in 2013, 4.4% in 2014, 3.3% in 2015, 3.5% in 2016, 2.2% in 2017, 2.7% in 2018, and 2.4% in 2019 (*p* = 0.0013; Figure 2A). The study spanned 143 hospitals in total. When comparing non‐surgical treatment rates among high‐volume (>200 cases, 19 hospitals, average: 3.02%, median: 3.3%, range: 0% to 8.56%), medium‐volume (100–199 cases, 15 hospitals, average: 3.81%, median: 2.04%, range: 0% to 30.0%), and low‐volume (1–99 cases, 109 hospitals, average: 7.16%, median: 0%, range: 0% to 100%) hospitals, significant differences were found (*p* < 0.0001; Figure 2B).

### 3.2 Patient characteristics

A total of 182 patients underwent primary RT, with some receiving additional chemotherapy. Among them, 145 patients, representing 80% of the total, completed primary definitive RT with a dosage of 6600 cGy or more (Figure 1). The characteristics of the study participants, categorized by their initial treatment type, are presented in Table 1. The group that received definitive RT exhibited a higher prevalence of recognized RFs compared to the group that underwent initial surgery (*p* < 0.0001). These included specific tumor subsites such as the floor of mouth [SMD −17.48], hard palate [SMD −21.87], and retromolar trigone [SMD −19.86]; older age [SMD −52.12]; stage II tumors [SMD −49.51]; and an elevated Charlson Comorbidity Index (CCI) [SMD −40.24]. For the purpose of this study, a weighted CCI was utilized.^10^ To balance these differences, PS matching was employed, which successfully achieved an adequate balance (SMD <10%) for all variables.

**TABLE 1:** General characteristics of patients with cT1−2N0M0 oral cavity squamous cell carcinoma based on initial surgery versus non‐surgical treatment (primary radiotherapy with a dosage of 6600 cGy or higher) before and after propensity score matching.

Characteristic (*n*, %)	Before propensity score matching				After propensity score matching		
	Initial surgery	Definitive Radiotherapy	*p*	SMD (%)	Initial surgery	Definitive radiotherapy	SMD (%)
	(*n* = 13,542)	(*n* = 145)			(*n* = 580)	(*n* = 145)	
Tumor subsite							
Lip (473, 3.5)	465 (3.4)	8 (5.5)	<0.0001	−10.09	38 (6.6)	8 (5.5)	4.35
Tongue (6026, 44)	4570 (43.4)	50 (34.5)		19.85	200 (34.5)	50 (34.5)	0.00
Gum (949, 6.9)	736 (7.0)	12 (8.3)		−5.12	41 (7.1)	12 (8.3)	−4.54
Mouth floor (565, 4.1)	431 (4.1)	12 (8.3)		−17.48	35 (6.0)	12 (8.3)	−8.70
Hard palate (213, 1.6)	144 (1.4)	8 (5.5)		−21.87	40 (6.9)	8 (5.5)	5.72
Buccal (4741, 34.6)	3716 (35.3)	41 (28.3)		13.88	168 (29.0)	41 (28.3)	1.53
Retromolar (500, 3.7)	386 (3.7)	12 (8.3)		−19.86	51 (8.8)	12 (8.3)	1.85
Other (220, 1.6)	178 (1.7)	2 (1.3)		1.90	7 (1.1)	2 (1.3)	−1.53
Sex							
Men (12,031, 87.9)	9297 (88.3)	128 (88.3)	0.8893	−1.17	518 (89.3)	128 (88.3)	3.28
Women (1656, 12.1)	1234 (11.7)	17 (11.7)		1.17	62 (10.7)	17 (11.7)	−3.28
Age (years)			<0.0001				
Mean ± SD	55.77 ± 11.70	62.39 ± 13.63	<0.0001	−52.12	61.70 ± 11.83	62.39 ± 13.63	−5.41
Clinical stage							
I (7570, 55.3)	6220 (59.1)	46 (31.7)	<0.0001	49.51	173 (29.8)	46 (31.7)	−4.11
II (6117, 44.7)	4311 (40.9)	99 (68.3)		−49.51	407 (70.2)	99 (68.3)	4.11
Charlson Comorbidity Index (mean ± SD)	0.83 ± 1.20	1.37 ± 1.47	<0.0001	−40.24	1.34 ± 1.51	1.37 ± 1.47	−2.01

Abbreviations: SD, standard deviation; SMD, standardized mean difference.

### 3.3 Pathological stages and occult metastasis rates in patients who received initial surgery

Among the 13,542 patients with cT1–2N0 OCSCC who underwent initial surgery, the distribution of final pathological stages was as follows: 45% were classified as pT1 (*n* = 6146), 42% as pT2 (*n* = 5661), 10% as pT3 (*n* = 1382), and 3% as pT4 (*n* = 353), according to the AJCC Staging Manual, eighth edition. In addition, 65% of these patients underwent prophylactic neck dissection. The nodal involvement was categorized into five groups: pNx, which represents no neck dissection (35%; *n* = 4716), pN0 (56%; *n* = 7570), pN1 (5%; *n* = 677), pN2 (3%; *n* = 404), and pN3 (1%; *n* = 175). When considering the overall pathological stages, the following distribution was observed: *p*‐stage I (44%; *n* = 5935), *p*‐stage II (37%, *n* = 5017), *p*‐stage III (13%; *n* = 1710), and *p*‐stage IV (7%; *n* = 880). Patients who did not undergo neck dissection (pNx) were recorded as pN0 when calculating the total stage. The rate of occult metastases for cT1–2N0 was 14.2% (1256 out of 8826). When broken down further, the occult metastasis rates for cT1N0 and cT2N0 tumors were 6.5% (170 out of 2621) and 17.5% (1086 out of 6205), respectively.

### 3.4 Five‐year survival rates

**FIGURE 3:**
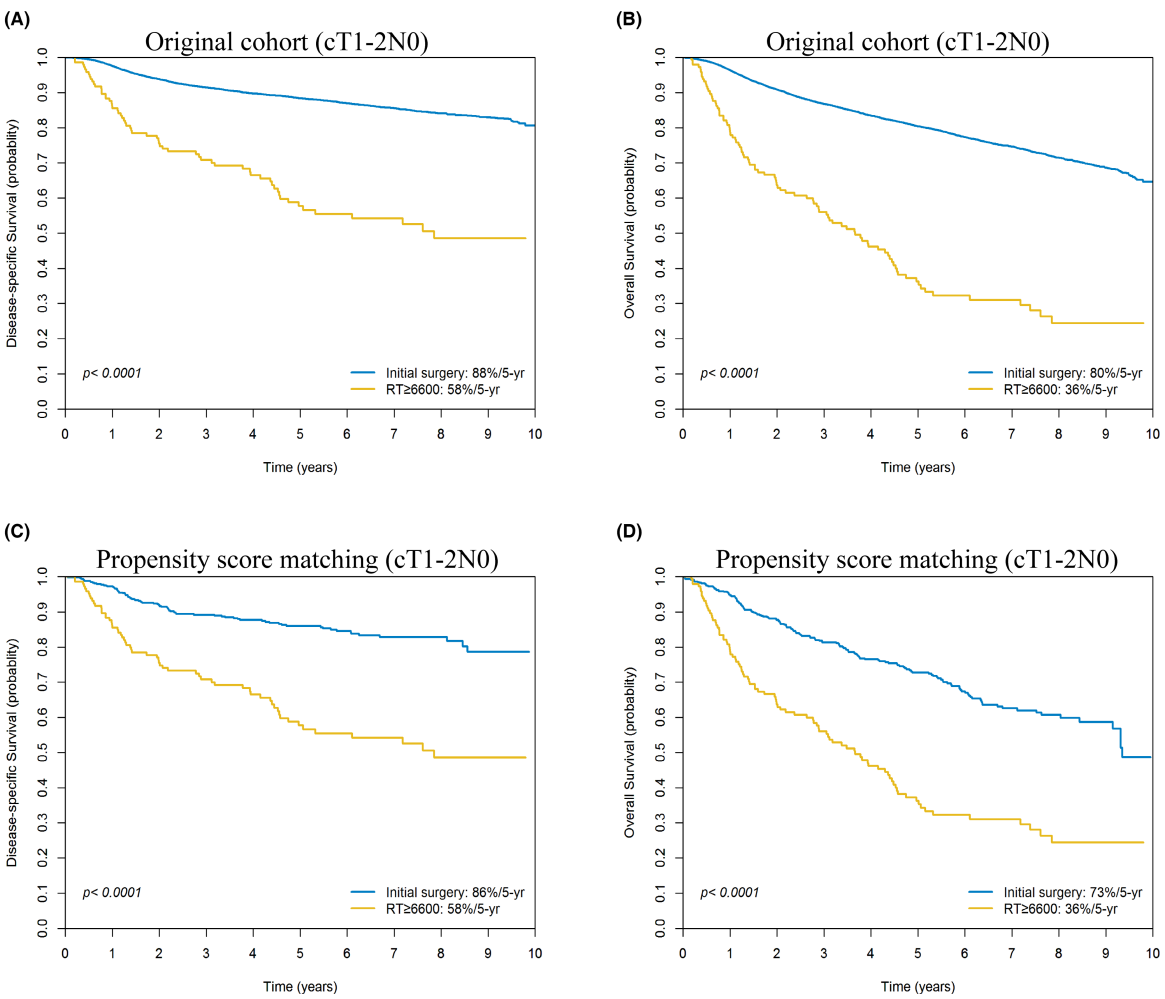
Kaplan–Meier plots of 5‐year disease‐specific survival and overall survival for cT1−2N0M0 patients who received initial surgery versus primary definite radiotherapy pre‐ (A, B) and post‐ (C, D) propensity score matching.

We observed 5‐year DSS and OS rates of 88% and 79%, respectively, across all participants. Before PS matching, the group that underwent initial surgery demonstrated superior 5‐year DSS (88% vs. 58%, *p* < 0.0001) and OS rates (80% vs. 36%, *p* < 0.0001) compared to the group that received definitive RT (Figure 3A,B). After PS matching, the initial surgery group continued to show higher 5‐year DSS (86% vs. 58%, *p* < 0.0001) and OS rates (73% vs. 36%, *p* < 0.0001) than the definitive RT group (Figure 3C,D).

Before PS matching, the 5‐year DSS (93% vs. 63%, *p* < 0.0001) and OS rates (86% vs. 43%, *p* < 0.0001) for patients with cT1N0M0 (*n* = 7570) who underwent initial surgery were significantly higher than those who received primary RT. After applying PS matching to balance the treatment groups, the 5‐year DSS remained the same at 93% versus 63% (*p* < 0.0001), whereas the OS rate slightly decreased to 80% versus 43% (*p* < 0.0001) for the surgery and RT groups, respectively.

Before PS matching, the 5‐year DSS and OS rates for cT2N0M0 patients (*n* = 6117) who underwent initial surgery were 86% and 77%, respectively, compared to 55% and 33% for those who received RT (both *p* < 0.0001). After PS matching, the 5‐year DSS and OS rates remained consistent at 84% and 70% for surgery, and 55% and 33% for primary RT, respectively (*p* < 0.0001).

### 3.5 Univariable and multivariable Cox regression analyses

Table 2 presents the outcomes of both univariable and multivariable analyses. After adjusting for potential confounders, multivariable analysis identified several factors that were independently linked to a decreased 5‐year DSS rate. These variables included treatment with primary RT (HR = 3.38, *p* < 0.0001), advanced age, a clinical stage II classification, and a high CCI. The RFs that were independently associated with a less favorable 5‐year OS were treatment with primary RT (HR = 3.35, *p* < 0.0001), male sex, advanced age, a clinical stage II classification, and a high CCI (Table 2). Interestingly, the results from the competing risk model analysis of DSS were consistent with those from the Cox proportional hazards regression analysis, particularly in the case of treatment with primary RT (subdistribution hazard ratio, [SHR] =3.35, *p* < 0.0001; Table 2).

**TABLE 2:** Univariable and multivariable analyses of risk factors for 5‐year disease‐specific and overall survival in the entire study cohort of patients with (*n* = 13,687) according to hazard ratio (HR, standard Cox regression) and subdistribution hazard ratio (SHR, competing risk).

	Disease‐specific survival								Overall survival			
	Univariable analysis		Stepwise multivariable analysis		UVA—Competing risk analysis		MVA—Competing risk analysis		Univariable analysis		Stepwise multivariable analysis	
	HR (95% CI)	*p*	HR (95% CI)	*p*	SHR (95% CI)	*p*	SHR (95% CI)	*p*	HR (95% CI)	*p*	HR (95% CI)	*p*
Treatment approach												
Initial surgery	1		1		1		1		1		1	
Primary RT with a dosage of 6600 cGy or higher	4.09 (3.17–5.28)	<0.0001	3.38 (2.61–4.37)	<0.0001	4.05 (3.12–5.26)	<0.0001	3.35 (2.57–4.37)	<0.0001	4.30 (3.50–5.28)	<0.0001	3.35 (2.73–4.12)	<0.0001
Tumor subsite												
Lip	1		‐		1		‐		1		‐	
Tongue	0.91 (0.70–1.18)	0.4763	‐	ns	0.91 (0.71–1.17)	0.4709	‐	ns	0.93 (0.76–1.14)	0.4689	‐	
Gum	0.94 (0.70–1.27)	0.6921	‐	ns	0.94 (0.70–1.26)	0.6784	‐	ns	1.03 (0.81–1.30)	0.8336	‐	
Mouth floor	0.88 (0.62–1.23)	0.4481	‐	ns	0.88 (0.63–1.23)	0.4419	‐	ns	1.22 (0.95–1.58)	0.1174	‐	
Hard palate	1.08 (0.71–1.64)	0.7310	‐	ns	1.09 (0.71–1.66)	0.6967	‐	ns	1.15 (0.83–1.60)	0.3868	‐	
Buccal	0.78 (0.60–1.01)	0.0582	‐	ns	0.78 (0.60–1.002)	0.0520	‐	ns	0.81 (0.66–0.998)	0.0474	‐	
Retromolar	1.01 (0.72–1.41)	0.9725	‐	ns	1.01 (0.72–1.40)	0.9674	‐	ns	1.12 (0.86–1.45)	0.4074	‐	
Other	0.82 (0.51–1.30)	0.3908	‐	ns	0.82 (0.52–1.29)	0.3942	‐	ns	0.93 (0.65–1.32)	0.6719	‐	
Sex												
Men	1.11 (0.96–1.30)	0.1700	‐	ns	1.12 (0.95–1.30)	0.1703	‐	ns	1.19 (1.06–1.35)	0.0039	1.29 (1.14–1.46)	<0.0001
Women	1		‐		1		‐		1		1	
Age (years)	1.014 (1.010–1.018)	<0.0001	1.010 (1.006–1.014)	<0.0001	1.014 (1.010–1.019)	<0.0001	1.010 (1.005–1.015)	<0.0001	1.030 (1.027–1.033)	<0.0001	1.025 (1.021–1.028)	<0.0001
Clinical stage												
I	1		1		1		1		1		1	
II	1.64 (1.49–1.80)	<0.0001	1.60 (1.45–1.76)	<0.0001	1.63 (1.48–1.80)	<0.0001	1.60 (1.45–1.76)	<0.0001	1.52 (1.41–1.63)	<0.0001	1.48 (1.37–1.59)	<0.0001
Charlson Comorbidity Index	1.12 (1.08–1.16)	<0.0001	1.09 (1.05–1.13)	<0.0001	1.12 (1.08–1.16)	<0.0001	1.09 (1.05–1.13)	<0.0001	1.26 (1.23–1.29)	<0.0001	1.21 (1.18–1.24)	<0.0001

Abbreviations: CI, confidence interval; HR, hazard ratio; RT, radiotherapy; SHR, subdistribution hazard ratio.

### 3.6 Five‐year survival rates: Comparison of different initial surgery and primary radiotherapy subgroups

Out of 13,542 patients diagnosed with OCSCC, 10895 (80.5%) underwent surgery alone, whereas 2647 (19.5%) received surgery followed by adjuvant therapy. The 5‐year DSS rates were 91% for the surgery‐only group and 78% for the group receiving surgery followed by adjuvant therapy (*p* < 0.0001). Similarly, the 5‐year OS rates for the two groups were 85% and 69%, respectively (*p* < 0.0001). The lower survival rates in the adjuvant therapy group are likely attributable to occult nodal metastases and adverse pathological RFs.

Among the 13,542 patients with OCSCC who had initial surgery, 4% (*n* = 562) had positive surgical margins, 45% (*n* = 6160) had close margins (less than 5 mm), 38% (*n* = 5119) had clear margins (5 mm or more), and for 13% (*n* = 1699), the margin status was unknown. Of the 145 patients who received definitive RT, 43% (*n* = 62) had RT alone, whereas 57% (*n* = 83) underwent RT combined with chemotherapy.

**FIGURE 4:**
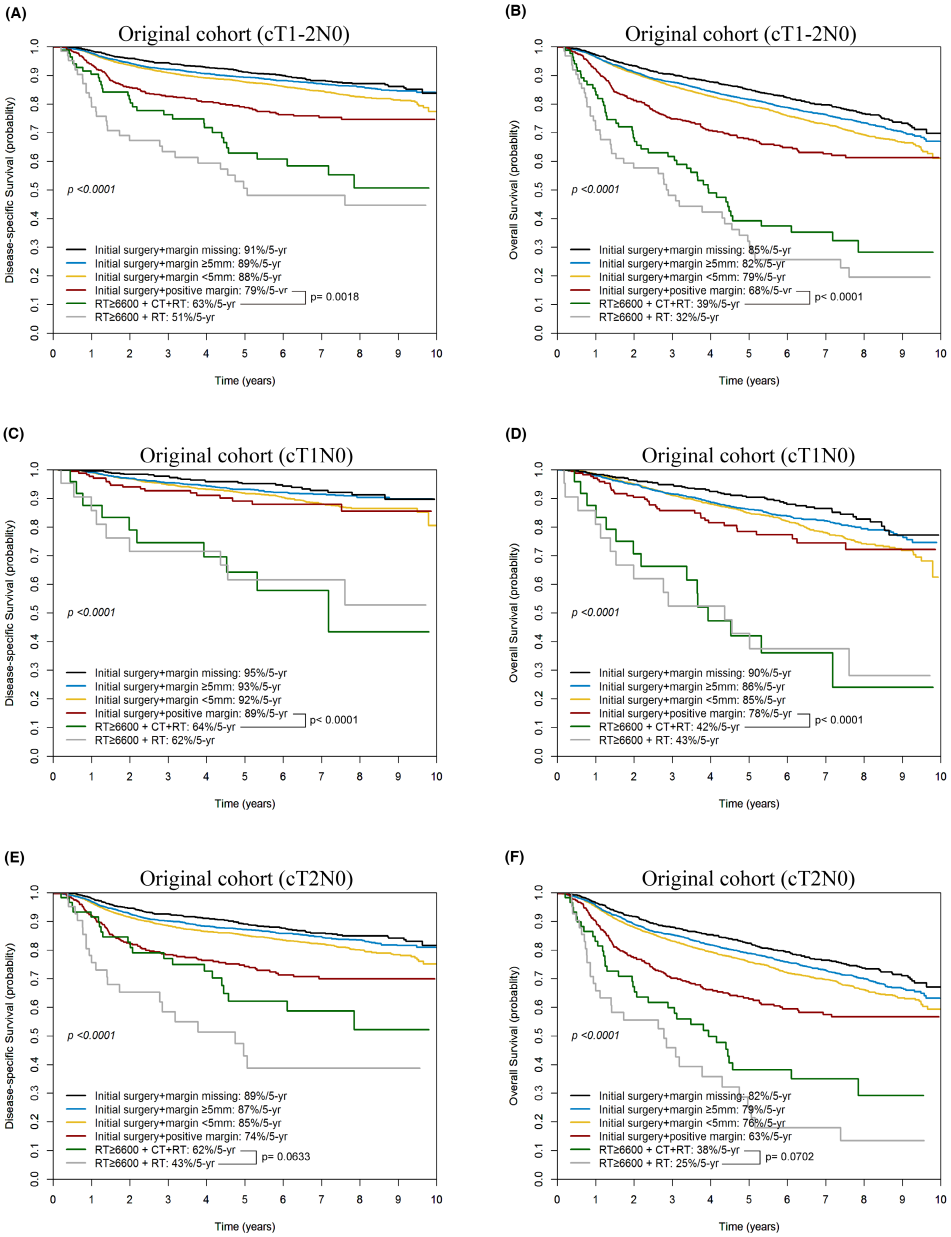
Kaplan–Meier plots of 5‐year disease‐specific survival and overall survival for cT1−2N0M0 patients in initial surgery subgroups (clear vs. close vs. positive margins) and primary RT subgroups (RT combined with chemotherapy vs. RT alone) (A, B); separate analyses for cT1N0M0 (C, D) and cT2N0M0 (E, F) patients.

In patients with cT1–2N0 tumors (*n* = 13,687), the 5‐year DSS (Figure 4A) and OS rates (Figure 4B) varied based on margin statuses and treatments. Patients with margins of 5 mm or more had a DSS rate of 89% and an OS rate of 82%. For those with margins less than 5 mm, the DSS and OS rates were 88% and 79%, respectively. Patients with positive margins had lower rates, with a DSS of 79% and an OS of 68%. When RT was combined with chemotherapy, the DSS and OS rates dropped to 63% and 39%, respectively. For patients who underwent RT alone, the rates were even lower, with a DSS of 51% and an OS of 32% (*p* < 0.0001) (Figure 4A,B). Notably, patients with positive margins had significantly higher 5‐year DSS and OS rates compared to those who received primary RT alone or in combination with chemotherapy.

When comparing patients who underwent initial surgery and had a positive margin to those who received RT and chemotherapy, the DSS rates were 79% and 63% respectively (*p* = 0.0018). Similarly, the OS rates were 68% for the surgery group and 39% for the RT plus chemotherapy group (*p* < 0.0001).

In the subset of cT1N0 patients (*n* = 7570), the 5‐year outcomes varied based on treatment and surgical margin. For patients with surgical margins of ≥5 mm, <5 mm, positive margins, those who received RT combined with chemotherapy, and those who received RT alone, the DSS rates were 93%, 92%, 89%, 64%, and 62%, respectively (*p* < 0.0001; Figure 4C). The corresponding OS rates were 86%, 85%, 78%, 42%, and 43%, respectively (*p* < 0.0001; Figure 4D). For the cT2N0 patient group (*n* = 6117), the 5‐year outcomes also varied based on treatment and surgical margin. The DSS rates for patients with surgical margins of ≥5 mm, <5 mm, positive margins, those who received RT combined with chemotherapy, and those who received RT alone were 87%, 85%, 74%, 62%, and 43%, respectively (*p* < 0.0001; Figure 4E). The corresponding OS rates were 79%, 76%, 63%, 38%, and 25%, respectively (*p* < 0.0001; Figure 4F).

Finally, the combination of RT and chemotherapy showed a nonsignificant trend toward improved 5‐year DSS and OS rates in patients with cT1−2N0 OCSCC, compared to those who received RT alone (Figure 4A,B). The impact of this combined treatment was more noticeable in cT2N0 patients than in cT1N0 patients. Specifically, for cT2N0 patients, the 5‐year DSS was 62% with combined treatment versus 43% with RT alone (*p* = 0.0633), and the 5‐year OS was 38% versus 25% (*p* = 0.0702; Figure 4C,D). For cT1N0 patients, the 5‐year DSS was 64% versus 62% (*p* = 0.8348), and the 5‐year OS was 42% versus 43% (*p* = 0.9783; Figure 4E,F). After PS matching, we observed similar survival trends (Figure S1A–F).

## 4 DISCUSSION

In this study, we found that Taiwanese patients diagnosed with cT1–2N0M0 OCSCC who underwent initial surgical treatment had significantly higher 5‐year DSS and OS rates compared to those who received RT. Multivariable analysis identified age, stage II disease, and CCI as independent RFs contributing to the 5‐year DSS and OS outcomes. Despite the tumor subsite not emerging as an independent prognostic factor, we included all variables in the PS matching process. Following PS matching, all parameters demonstrated a SMD of less than 10% between the two treatment groups, indicating no significant differences. Notably, the intergroup disparities in 5‐year DSS and OS rates remained significant post‐PS matching, with rates of 86% versus 58% and 73% versus 36%, respectively. Of particular note, among the RFs examined, the initial treatment approach (initial surgery compared to definitive RT) showed the highest HRs. For DSS, the HR was 3.40, whereas for OS, it was 3.35. This suggests that the initial treatment approach significantly influences survival outcomes.

Surgery is generally the preferred initial treatment for OCSCC, whereas RT is frequently suggested as an adjuvant treatment after surgery or as an alternative for recurring tumors. Our study found a lower 5‐year DSS rate in the group treated with definitive RT compared to the group that underwent initial surgery (Figure 3A,C). Moreover, the difference in 5‐year OS between the two groups was even more significant (Figure 3B,D). This disparity might be due to the long‐term morbidity resulting from complications related to the treatment.

The NCCN guidelines suggest a definitive RT dose of 6600–7000 cGy for cT1−2N0M0 OCSCC.^1^ However, many radiation oncologists opt for doses of 7000 cGy or higher to manage gross tumor disease. There is ongoing research suggesting that overcoming the relative radioresistance of OCSCC may require more aggressive strategies such as interstitial brachytherapy, dose escalation, or larger fraction sizes.^11^ However, these intensive treatments could lead to severe long‐term morbidity and a decrease in the patient's quality of life.

Three nationwide studies have been published on patients with cT1−2N0M0 OCSCC, revealing a 30% difference in survival rates between those who underwent primary surgery and those who received definitive RT.^2^, ^3^, ^4^ However, it is crucial to highlight that the criteria for primary RT doses varied across these studies. The study by Sowder et al.^2^ conducted from 1998 to 2008 with 8100 participants, did not document RT arm doses. Ellis et al. study, conducted from 2004 to 2013 with 20,779 participants, categorized RT arm doses as follows: less than 4000 cGy (7%), 4000–5999 cGy (14%), 6000–6600 cGy (21%), and more than 6600 cGy (59%).^3^ Liu et al.'s study, conducted from 2006 to 2015 with 1648 participants after PS matching, only reported a median RT arm dose of 7000 cGy, without specifying the range.^4^ Given these variations, further investigation is needed to clarify the actual survival difference between patients who underwent initial surgery and those who received definitive RT (≥6600 cGy).

According to the guidelines set by the NCCN, definitive RT is suggested as an alternative treatment for cases of OCSCC that are classified as cT1−2N0M0. On the other hand, chemotherapy is not recommended for these cases. This is reflected in the studies conducted by Ellis et al.^3^ and Liu et al.,^4^ where the researchers focused exclusively on patients who underwent RT, deliberately excluding those who received a combination of RT and chemotherapy. In a separate study by Sowder et al.^2^ there was no information provided about chemotherapy. It is noteworthy that, in clinical practice, certain radiation oncologists opt for chemoradiotherapy when treating cT2N0 tumors. This approach is primarily due to a greater tumor burden and increased incidence of occult metastases in the neck, as compared to cT1N0 tumors. Furthermore, a higher prevalence of occult metastases has been observed in the tongue subsite relative to other locations. El‐Naaj et al.'s study provides evidence for this, revealing a higher incidence of occult metastasis (34%) in cases of cT1−2N0 tongue squamous cell carcinoma.^12^ This finding underscores the necessity for postoperative RT and chemotherapy, primarily driven by the presence of occult metastasis.

In our study, we combined data from patients who underwent RT alone and those who received both RT and chemotherapy. The findings revealed that the combined treatment led to better 5‐year DSS and OS rates, particularly in cT2N0 patients compared to cT1N0 patients. Previous studies have indicated that occult metastases occur in approximately 10%–15% of cT1N0 OCSCC cases and over 20% of cT2N0 OCSCC cases.^13^, ^14^ Consistent with our prior research,^15^ the rates of occult metastases observed in this study (cT1N0 [6.5%]; cT2N0 [17.5%]) were lower than the reported averages in the literature. In light of these findings, consistently incorporating chemotherapy into the RT regimen could potentially enhance locoregional control in cT2N0 OCSCC patients.^16^

In the present study, 19.5% of patients from the initial group underwent adjuvant therapy, a figure that aligns with the NCDB database study where 19.4% (3836 out of 19,823) of patients received both initial surgery and adjuvant therapy. The group that underwent initial surgery alone demonstrated the highest 5‐year survival rate (*n* = 10,895, DSS = 91%, OS = 83%). This is likely due to the fact that the majority of these patients did not present with hidden nodal metastasis or adverse pathologic RFs. Conversely, the group that received RT alone (*n* = 62) exhibited the lowest survival rates (5‐year DSS = 51%, 5‐year OS = 32%), possibly due to a higher incidence of hidden nodal metastases in cT2N0 patients. Given the small size of the RT alone group, we did not further investigate the comparison between the initial surgery alone group and the RT alone group.

The NCCN guidelines recommend that patients with OCSCC who have undergone resection and exhibit positive margins and lymph node extracapsular extension (ENE) should receive postoperative concurrent chemoradiotherapy.^1^ Our previous research has shown that a positive surgical margin is an independent risk factor for patient outcomes in cases with ENE.^17^ In this study, we found that initial surgery, even with positive margins, resulted in better outcomes than definitive RT for most cT1−2N0M0 OCSCC patients, irrespective of additional chemotherapy. Achieving clear surgical margins is crucial for improved outcomes. Our prior nationwide study demonstrated that clear margins reduce the need for adjuvant RT, thereby enhancing the patient's quality of life.^18^

The present research identified a higher number of older patients, as well as those with tumors located in the hard palate or retromolar trigone, and T2 tumors, in the RT group. This preference for non‐surgical treatment could be attributed to various factors, including the increased risk of complications in elderly patients, the complexities involved in postoperative flap reconstruction for certain regions, and the extensive reconstruction required for T2 tumors compared to T1 tumors. These findings are consistent with the results reported by Sowder et al.^2^ which also indicated a higher prevalence of older patients and those with tumors in challenging locations such as the hard palate and retromolar trigone, as well as a higher prevalence of T2 tumors, in the primary RT group. Similarly, Ellis et al. found a greater prevalence of patients aged over 70 years and T2 tumors in the primary RT group.^3^ Their study also revealed that the RT group had more patients with oral tumors located in areas other than the tongue.^3^ Furthermore, they showed that the RT group included a higher number of patients during the early years of treatment.^3^ A significant decline in primary RT rates was observed over the years, decreasing from 8.7% in 2004–2005 to 2.7% in 2012–2013. We initially recorded a non‐surgical rate of 3.3%, which significantly dropped from 4.6% in 2011 to 2.4% in 2019. Ellis et al.'s study also found that the RT group had a higher percentage of patients treated at low‐volume facilities.^3^ When primary RT treatment rates were categorized by the annual number of cases per hospital, the following distribution was found: 8.6% for one case, 5.0% for 2–4 cases, 2.7% for 5–10 cases, and 1.3% for more than 10 cases. In our study, the initial non‐surgical rate was 3.3% (469 out of 13,542), while the final definitive RT group constituted only 1.1% (145 out of 13,542) of the total cases. Consequently, our primary focus was on the original non‐surgical group within the hospital data. In Taiwan, OCSCC is a common type of cancer, primarily due to the prevalent habit of betel nut chewing.^19^ In this study, we categorized hospitals into three groups based on the number of OCSCC cases they manage: low‐volume hospitals (1–99 cases), medium‐volume hospitals (100–199 cases), and high‐volume hospitals (over 200 cases). Our analysis revealed a statistically significant difference in non‐surgical treatment rates between low‐volume hospitals and medium‐ or high‐volume hospitals (mean: 7.16% vs. 3.81% and 3.02%). Notably, at 11 institutions, including one high‐volume, two medium‐volume, and eight low‐volume hospitals, the rate of initial non‐operative procedures exceeded the outlier (Figure 2B).

In summary, primary surgery emerges as the favored treatment for OCSCC across healthcare facilities in Taiwan, with a noticeable shift away from initial non‐surgical interventions. Despite this, non‐surgical approaches continue to be prevalent in certain centers. A thorough evaluation of treatment protocols in these facilities could contribute to achieving the best possible outcomes. Furthermore, it might be beneficial to consider relocating patients who are good candidates for surgery to hospitals that frequently perform such procedures.

To our knowledge, this is the first nationwide study comparing initial surgery and RT at ≥6600 cGy for patients with cT1−2N0 OCSCC patients. Our findings suggest that cT2N0 patients who underwent definitive RT combined with chemotherapy had improved survival rates. However, the statistical significance of this improvement was marginal when compared to those who received definitive RT alone. There are, however, limitations to our study. The TCRD, which we used for our research, does not include information on chemotherapy prescriptions. This lack of data prevents us from fully understanding the role of chemotherapy in definitive RT. In addition, our study sample was drawn from a region where betel nut chewing is prevalent. This habit is associated with a lower incidence of occult metastases compared to areas where betel nut use is less common.^20^ Furthermore, chronic areca nut exposure has been linked to chemo‐radioresistance and cancer stemness conversion, which may cause treatment failures.^21^ These factors may limit the applicability of the study findings to Western populations. Finally, the study dataset lacks potentially significant confounders that could impact treatment selection and outcomes. These include marital status, social support, socioeconomic status, body mass index, performance status, sarcopenia, and psychiatric comorbidities.

In summary, our research revealed that only 1.1% of cT1−2N0M0 OCSCC patients in Taiwan underwent definitive RT, resulting in a 30% survival difference between the initial surgery and RT groups. Interestingly, patients with positive surgical margins exhibited superior survival rates post‐surgery compared to the RT group. Given the substantial survival difference between initial surgery and RT groups, it may be worth reconsidering the inclusion of RT as an initial alternative treatment in the NCCN guidelines.

## AUTHOR CONTRIBUTIONS

**Chien‐Yu Lin:** Conceptualization (lead); data curation (lead); formal analysis (lead); investigation (lead); methodology (lead); project administration (lead); resources (lead); software (lead); supervision (lead); validation (lead); visualization (lead); writing – original draft (lead); writing – review and editing (lead). **Wen‐Cheng Chen:** Conceptualization (lead); data curation (lead); formal analysis (lead); investigation (lead); methodology (lead); project administration (lead); resources (lead); software (lead); supervision (lead); validation (lead); visualization (lead); writing – original draft (lead); writing – review and editing (lead). **Yu‐Wen Wen:** Data curation (equal); formal analysis (equal); funding acquisition (lead); investigation (equal); methodology (equal); resources (equal); software (equal); supervision (equal); validation (equal); writing – review and editing (equal). **Kang‐Hsing Fan:** Data curation (equal); formal analysis (equal); investigation (equal); methodology (equal); resources (equal); software (equal); supervision (equal); validation (equal); writing – review and editing (equal). **Jin‐Ching Lin:** Data curation (equal); formal analysis (equal); investigation (equal); methodology (equal); resources (equal); software (equal); supervision (equal); validation (equal); writing – review and editing (equal). **Shu‐Hang Ng:** Data curation (equal); formal analysis (equal); investigation (equal); methodology (equal); resources (equal); software (equal); supervision (equal); validation (equal); writing – review and editing (equal). **Yao‐Te Tsai:** Data curation (equal); formal analysis (equal); investigation (equal); methodology (equal); resources (equal); software (equal); supervision (equal); validation (equal); writing – review and editing (equal). **Shu‐Ru Lee:** Data curation (equal); formal analysis (equal); investigation (equal); methodology (equal); resources (equal); software (equal); supervision (equal); validation (equal); writing – review and editing (equal). **Chung‐Jan Kang:** Data curation (equal); formal analysis (equal); investigation (equal); methodology (equal); resources (equal); software (equal); supervision (equal); validation (equal); writing – review and editing (equal). **Li‐Yu Lee:** Data curation (equal); formal analysis (equal); investigation (equal); methodology (equal); resources (equal); software (equal); supervision (equal); validation (equal); writing – review and editing (equal). **Chih‐Yen Chien:** Data curation (equal); formal analysis (equal); investigation (equal); methodology (equal); resources (equal); software (equal); supervision (equal); validation (equal); writing – review and editing (equal). **Chun‐Hung Hua:** Data curation (equal); formal analysis (equal); investigation (equal); methodology (equal); resources (equal); software (equal); supervision (equal); validation (equal); writing – review and editing (equal). **Cheng Ping Wang:** Data curation (equal); formal analysis (equal); investigation (equal); methodology (equal); resources (equal); software (equal); supervision (equal); validation (equal); writing – review and editing (equal). **Tsung‐Ming Chen:** Data curation (equal); formal analysis (equal); investigation (equal); methodology (equal); resources (equal); software (equal); supervision (equal); validation (equal); writing – review and editing (equal). **Shyuang‐Der TERNG:** Data curation (equal); formal analysis (equal); investigation (equal); methodology (equal); resources (equal); software (equal); supervision (equal); validation (equal); writing – review and editing (equal). **Chi‐Ying Tsai:** Data curation (equal); formal analysis (equal); investigation (equal); methodology (equal); resources (equal); software (equal); supervision (equal); validation (equal); writing – review and editing (equal). **Hung‐Ming Wang:** Data curation (equal); formal analysis (equal); investigation (equal); methodology (equal); resources (equal); software (equal); supervision (equal); validation (equal); writing – review and editing (equal). **Chia‐Hsun Hsieh:** Data curation (equal); formal analysis (equal); investigation (equal); methodology (equal); resources (equal); software (equal); supervision (equal); validation (equal); writing – review and editing (equal). **Chih‐Hua Yeh:** Data curation (equal); formal analysis (equal); investigation (equal); methodology (equal); resources (equal); software (equal); supervision (equal); validation (equal); writing – review and editing (equal). **Chih‐Hung Lin:** Data curation (equal); formal analysis (equal); investigation (equal); methodology (equal); resources (equal); software (equal); supervision (equal); validation (equal); writing – review and editing (equal). **Chung‐Kan Tsao:** Data curation (equal); formal analysis (equal); investigation (equal); methodology (equal); resources (equal); software (equal); supervision (equal); validation (equal); writing – review and editing (equal). **Nai‐Ming Cheng:** Data curation (equal); formal analysis (equal); investigation (equal); methodology (equal); resources (equal); software (equal); supervision (equal); validation (equal); writing – review and editing (equal). **Tuan‐Jen Fang:** Data curation (equal); formal analysis (equal); investigation (equal); methodology (equal); resources (equal); software (equal); supervision (equal); validation (equal); writing – review and editing (equal). **Shiang‐Fu Huang:** Data curation (equal); formal analysis (equal); investigation (equal); methodology (equal); resources (equal); software (equal); supervision (equal); validation (equal); writing – review and editing (equal). **Li‐Ang Lee:** Data curation (equal); formal analysis (equal); investigation (equal); methodology (equal); resources (equal); software (equal); supervision (equal); validation (equal); writing – review and editing (equal). **Ku‐Hao Fang:** Data curation (equal); formal analysis (equal); investigation (equal); methodology (equal); resources (equal); software (equal); supervision (equal); validation (equal); writing – review and editing (equal). **Yu‐Chien Wang:** Data curation (equal); formal analysis (equal); investigation (equal); methodology (equal); resources (equal); software (equal); supervision (equal); validation (equal); writing – review and editing (equal). **Wan‐Ni Lin:** Data curation (equal); formal analysis (equal); investigation (equal); methodology (equal); resources (equal); software (equal); supervision (equal); validation (equal); writing – review and editing (equal). **Li‐Jen Hsin:** Data curation (equal); formal analysis (equal); investigation (equal); methodology (equal); resources (equal); software (equal); supervision (equal); validation (equal); writing – review and editing (equal). **Tzu‐Chen Yen:** Data curation (equal); formal analysis (equal); investigation (equal); methodology (equal); resources (equal); software (equal); supervision (equal); validation (equal); writing – review and editing (equal). **Chun‐Ta Liao:** Conceptualization (lead); data curation (lead); formal analysis (lead); investigation (lead); methodology (lead); project administration (lead); resources (lead); software (lead); supervision (lead); validation (lead); visualization (lead); writing – original draft (lead); writing – review and editing (lead).

## CONFLICT OF INTEREST STATEMENT

The authors declare that there are no conflicts of interest that could potentially influence the presentation or interpretation of the research findings in this study.

## FUNDING INFORMATION

This study was financially supported by the Chang Gung Medical Research Program through grants CMRPD1H0521 and BMRPC55.

## ETHICS STATEMENT

The Chang Gung Memorial Hospital's Ethics Committee granted approval for the study (reference number: 201801398B0A3).

## PATIENT CONSENT STATEMENT

Given the retrospective nature of this study, which involved the analysis of pre‐existing data, the Ethics Committee at Chang Gung Memorial Hospital determined that obtaining written informed consent from the participants was not necessary.

## Supporting information


Figure S1.

## Data Availability

The availability of data used in this study is subject to third‐party restrictions imposed by the Health and Welfare Data Center of the Taiwanese Ministry of Health and Welfare (http://dep.mohw.gov.tw/DOS/), in accordance with the “Personal Information Protection Act.” Despite these limitations, the authors were granted a license to utilize the data for the purposes of this research. Access to the datasets generated and analyzed during this study can be provided by the corresponding author upon reasonable request. However, this is contingent on obtaining formal permission from the Taiwanese Ministry of Health and Welfare.
