# Supplementary figures and images for: Comparing the clinical outcomes of initial surgery and primary definitive radiotherapy with a dosage of 6600 cGy or higher in cT1−2N0M0 oral cavity squamous cell carcinoma: A nationwide cohort study
Source: Cancer Med. 2024 May 21;13(10):e7127. doi: 10.1002/cam4.7127 (PMC11106645; doi:10.1002/cam4.7127)

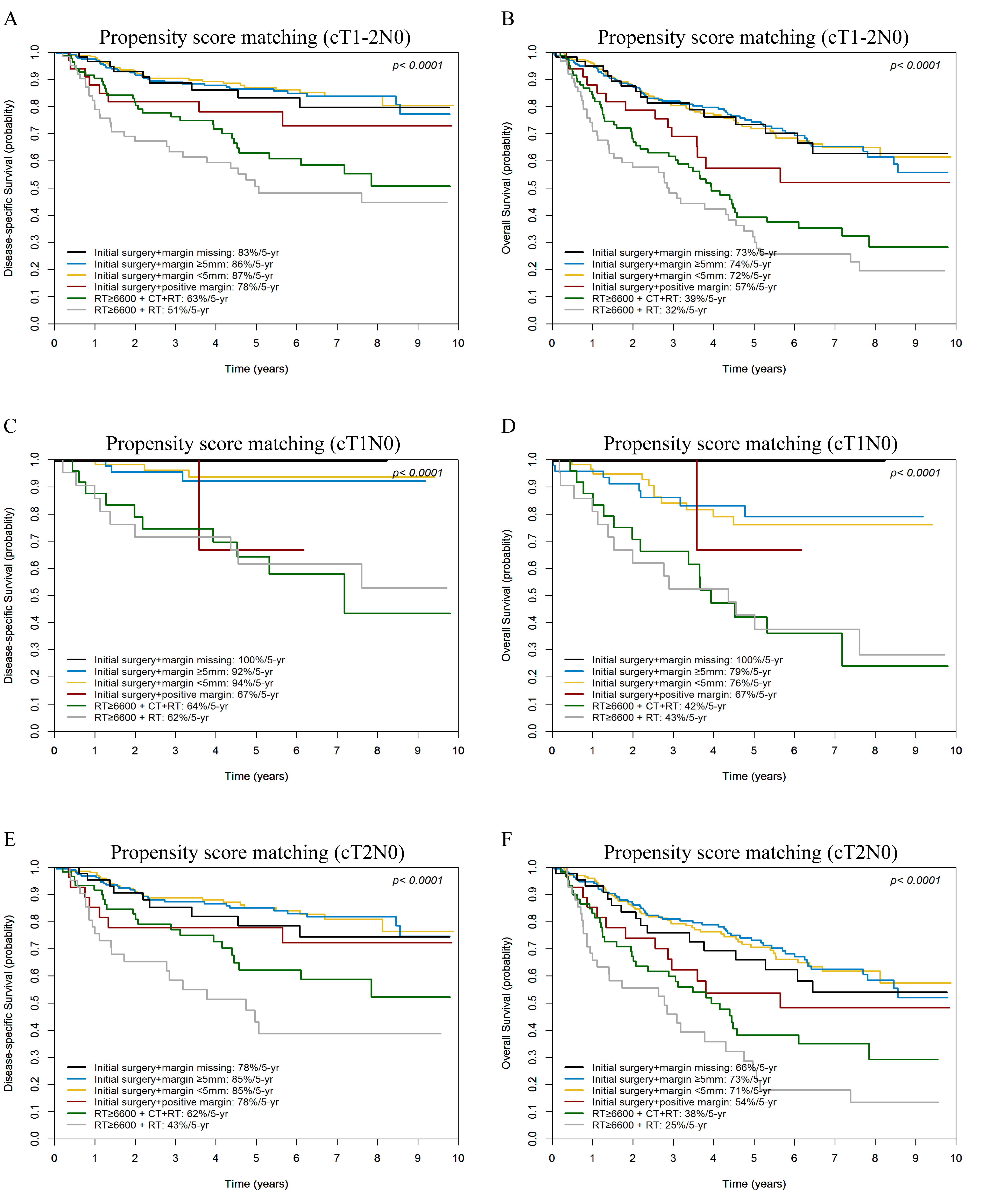

Supplement: Supplementary file 1 — Figure S1. [file CAM4-13-e7127-s001.tif]
